# Comparison of in-office and at-home tooth-whitening products cytotoxicity

**DOI:** 10.1080/07853890.2021.1895580

**Published:** 2021-09-28

**Authors:** Kristel Pitz, Susana Bandarra, Paulo Mascarenhas, Ana Clara Ribeiro, Ana Azul, Madalena Salema-Oom, Isabel Barahona

**Affiliations:** a Laboratório de Biologia Moleculare, Instituto Universitário Egas Moniz (IUEM), Caparica, Portugal; b Laboratório de Biomateriais do Centro de Investigação Interdisciplinar Egas Moniz (CiiEM), Instituto Universitário Egas Moniz (IUEM), Caparica, Portugal

## Abstract

**Introduction:**

Bleaching teeth to have a whiter and bright smile is a popular trend and currently one can do it at home, without technical support from dentists. However, whitening products are not innocuous and the European legislation is clear limiting the content in hydrogen peroxide (H_2_O_2_), present or released, to 6%, still, products containing up to 40% of H_2_O_2_ are commercially available. In fact, the contact time of live tissues with H_2_O_2_ agents is usually small, which may restrict side effects, but on the other hand, successively applications as well as at-home careless applications may be harmful. This work aims to verify how whitening products commercialised in Portugal impact on fibroblasts viability.

**Materials and methods:**

We used fibroblasts, the main cells of both pulp and gingivae that contact with whitening gels. Mouse embryo fibroblasts (NIH/3T3) were incubated with serial dilutions of Opalescence PF boost 40%, Opalescent PF 16%, Opalescent PF 10% from Ultradent (USA) and Bbryance 0.095% (France) in culture medium for 1-hour. After that period, the culture medium was removed and cell viability was determined using MTT assay.

**Results:**

Our results showed a huge decrease in fibroblasts viability after exposure to both product types: containing H_2_O_2_ or carbamide peroxide. To achieve conditions considered non-toxic, i.e. showing a reduction in cell viability <30% [1], it was necessary to dilute whitening products at least 100- to 62,500-fold, down to 0.0001–0.0004% H_2_O_2_, as shown in Table 1.

**Discussion and conclusions:**

Although we cannot extrapolate this effect directly to human teeth, because the concentration of H_2_O_2_ arriving at the pulp depends on diffusion through dentinal tubules, our observations are of great concern in particular for gingivae health. Moreover, the whitening products sold for at-home use are as cytotoxic as the in-office product to be applied under the supervision of the dental professional independently of the product type. Since we found that at-home products have similar toxicities, we anticipated that the BBRYANCE gel will induce less severe effects due to his lower H_2_O_2_ concentration.

**Table 1.** Necessary dilutions to achieved non-toxic conditions (viability >70%).

Utilisation	Product /Whitening agent	Equivalents in H_2_O_2_ in the product (%)	Product dilution	Predicted [H_2_O_2_] to achieve 70% viability
In-office	Opalescent PF boost 40%/ hydrogen peroxide	40	1/62,500	0.0001
At-home	Opalescent PF 16% / carbamide peroxide	5.8	1/37,500	0.0001
	Opalescent PF 10% / carbamide peroxide	3.6	>1/1500	0.0004
	BBRYANCE 0.095% / hydrogen peroxide	0.095	1/100	0.0002
